# Advances in the study of the glymphatic system and aging

**DOI:** 10.1111/cns.14803

**Published:** 2024-06-17

**Authors:** Ying Xiong, Qingying Yu, Haimei Zhi, Huiyuan Peng, Mingjun Xie, Renjun Li, Kejian Li, Yuexiang Ma, Peng Sun

**Affiliations:** ^1^ School of Traditional Chinese Medicine Shandong University of Traditional Chinese Medicine Jinan China; ^2^ Guangdong Provincial Key Laboratory of Translational Cancer Research of Chinese Medicines, Joint International Research Laboratory of Translational Cancer Research of Chinese Medicines, International Institute for Translational Chinese Medicine, School of Pharmaceutical Sciences Guangzhou University of Chinese Medicine Guangzhou China; ^3^ Qilu Hospital of Shandong University Jinan China; ^4^ Department of Rehabilitation Zhongshan Hospital of Traditional Chinese Medicine Zhongshan China; ^5^ Department of Psychiatry Jinan Mental Health Center Jinan China; ^6^ Innovative Institute of Chinese Medicine and Pharmacy Shandong University of Traditional Chinese Medicine Jinan China

**Keywords:** aging, glymphatic system, immunosenescence, neurodegenerative diseases

## Abstract

The glymphatic system is cerebrospinal fluid–brain tissue fluid exchange flow mediated by aquaporin‐4 (AQP4) on the end feet of astrocytes for a system, which is capable of rapidly removing brain metabolites and thus maintaining brain homeostasis, and is known as the central immune system. Dysfunction of the glymphatic system causes accumulation of misfolded and highly phosphorylated proteins (amyloid‐β and Tau proteins), which destabilizes the proteins, and the body's neuroinflammatory factors are altered causing aging of the immune system and leading to neurodegenerative diseases. Damage to the glymphatic system and aging share common manifestations, as well as unstudied biological mechanisms that are also linked, such as mitochondria, oxidative stress, chronic inflammation, and sleep. In this paper, we first summarize the structure, function, and research methods of the glymphatic system and the relationship between the glymphatic system and the peripheral immune system, and second, sort out and summarize the factors of the glymphatic system in removing metabolites and resolving aging‐related diseases and factors affecting aging, to explore its related biological mechanisms, and moreover, to provide a new way of thinking for treating or intervening aging‐related diseases.

## Graphical abstract

Structure and function of the glymphatic system. The relationship between the glymphatic system and peripheral immunity. Physiopathologic manifestations of the glymphatic system and aging. Mechanisms of glymphatic system affecting aging.

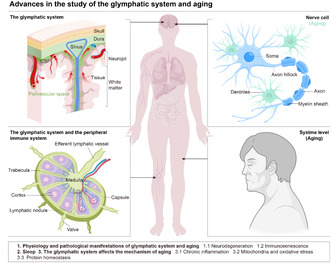

## 1 INTRODUCTION

Aging is an activity in the process of the occurrence and development of life and is a process in which the organism loses or even deteriorates from its constituent substances, and organizational structures to physiological functions.^1^ Aging has obvious changes at both the cellular and systemic levels of the organism, and cellular aging is mainly characterized by cell proliferation arrest, apoptosis resistance, and complex aging‐related secretory phenotypes.^2^ Systemic senescence is mainly the result of a stagnation of cell proliferation, apoptosis resistance, and complex senescence‐related secretory phenotype. Systemic senescence is characterized by impaired functioning between systems to maintain the organism. The most obvious phenomena caused by aging are immune decline^3^ and neurodegenerative lesions.^4^

It has been reported that aging is related to the normal functioning of the glymphatic system.^5^, ^6^ The central nervous system (CNS) has long been considered an “immune‐exempt” organ that lacks lymphatic vessels to transport immune cells.^7^ However, recent studies have demonstrated the presence of lymphatic vessels in the dura mater of humans and other animals.^8^, ^9^ These meningeal lymphatic vessels continuously drain fluids and molecules from the CNS to the periphery by connecting to the deep cervical lymph nodes (dCLNs) and play an important role in the active transport of immune cells.^8^, ^10^, ^11^ Changes in the glymphatic system have been reported to influence disease progression in aging‐associated neurodegenerative and neurologic‐related diseases, including Alzheimer's disease (AD)^12^, ^13^, ^14^ Parkinson's disease (PD),^15^, ^16^ traumatic brain injury (TBI),^17^, ^18^, ^19^ and cognitive impairment due to depression,^20^ and diabetic cognitive impairment.^21^ The glymphatic system is primarily dependent on the astrocytic lymphatic system, on which aquaporin 4 (AQP4), to drive the perivascular pathway of cerebrospinal fluid (CSF) throughout the brain to deliver nutrients and neuroactive substances, removes endogenous and exogenous metabolites and maintain homeostasis,^22^ thereby maintaining homeostasis in the brain. The adult brain removes approximately 7 g of waste protein per day.^23^ The majority of large protein molecules and solutes are cleared through this pathway. The deposition of abnormal proteins such as amyloid‐β (Aβ), tau, and α‐synuclein (α‐syn) is an important pathological feature in the development of neurodegenerative diseases, and the removal of these core disease‐causing substances is the key to preventing or slowing down the progression of the disease.^16^, ^24^, ^25^ The removal of these core pathogenic substances is the key to stopping or delaying disease progression. The glymphatic system is the main pathway to eliminate these proteins. It has been observed that early in the pathogenesis of AD, a reduction in glymphatic system transport occurs prior to Aβ deposition.^26^

Therefore, in this paper, the structure and function of the glymphatic system and its research progress in aging‐related diseases will be sorted out, and the common biological mechanisms of the glymphatic system and aging will be elaborated, in the hope that it can give more possibilities to the glymphatic system in the study of aging and provide new therapeutic strategies for the clinical treatment and intervention of aging‐related diseases.

## 2 GLYMPHATIC SYSTEM

### 2.1 Structure and composition

**FIGURE 1:**
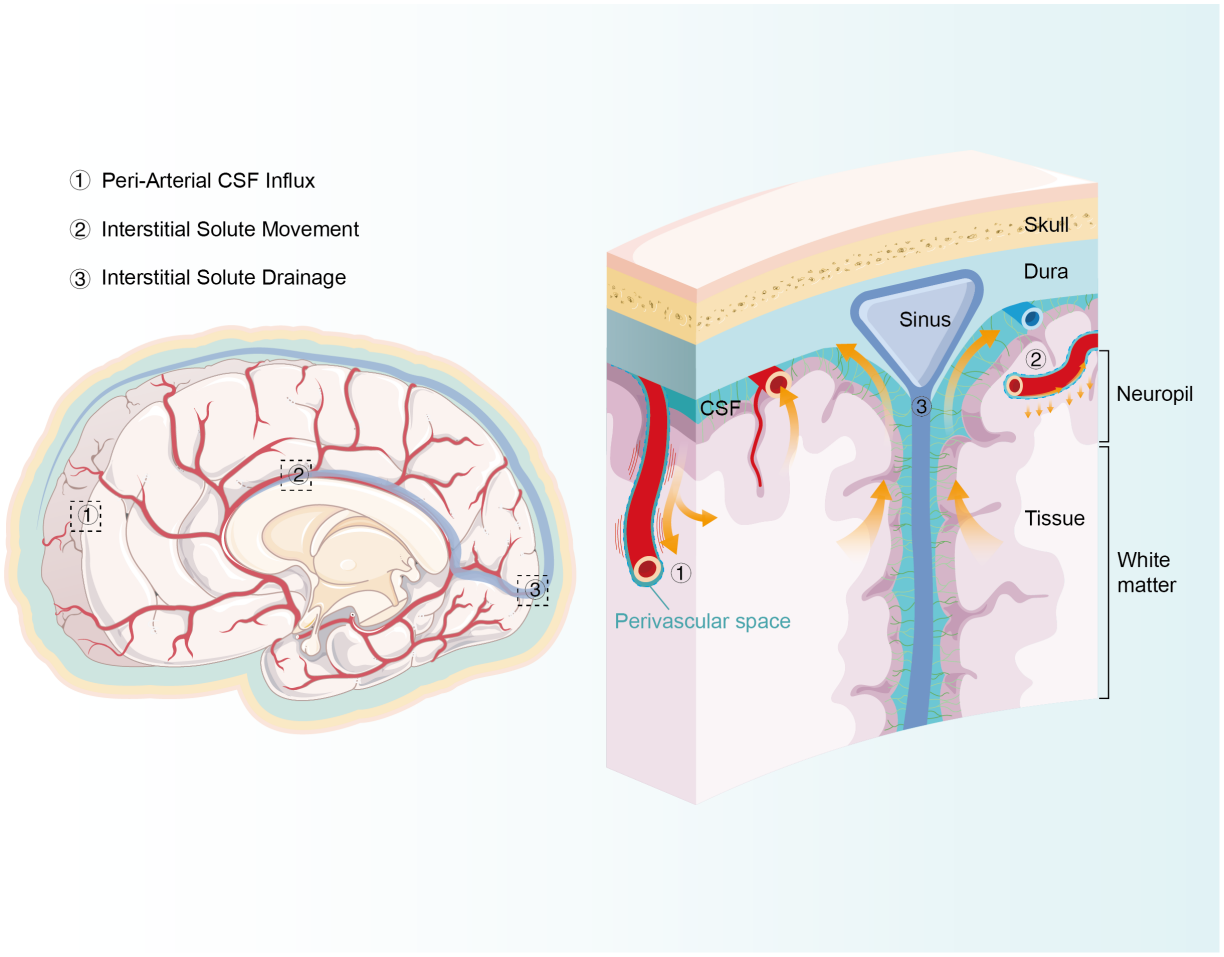
Schematic diagram of the glymphatic system. (1) Driven by arterial pulsation, cerebrospinal fluid flows into the brain tissue through the perivascular pathway around the artery. The large amount of flow around the blood vessels and the clearance of the mesenchymal solute depend on the astrocyte water channel AQP4, which is located at the end of the astrocyte around the blood vessel. (2) Interstitial solute movement occurs through the combined action of diffusion and flow. Advection is fastest along special anatomical pathways, including parenchymal perivascular spaces and white matter tracts, and supports the movement of macromolecular solutes. Diffusion dominates the movement of small molecules, especially within wider gaps. (3) Solutes in the interstitial are drained from the parenchyma along the white matter tract, the veins flow to the sinusoidal cisterna ventriculi, and the solutes are absorbed into the meningeal lymphatic vessels, discharged through the dural arachnoid particles, or cleared along the cranial or spinal nerve sheath.

The glymphatic system is a highly organized fluid transport system that originates from the cerebrospinal fluid (CSF) in the subarachnoid space, enters the brain along the periarterial vascular space, and AQP4 on the end feet of the astrocytes enters the interstitial fluid (ISF) and thus undergoes a solute exchange, and then the mixing of the neurofibrillary tangles are cleared to the meninges and cervical lymph nodes, thus entering the periphery.^27^, ^28^ Its transport mainly consists of the inward flow of peri‐arterial cerebrospinal fluid, the movement of interstitial solutes, and along the periventricular interstitium (Figure 1).^29^

### 2.2 Research methods of the glymphatic system

The research approach to the glymphatic system involves a variety of techniques and experimental designs aimed at revealing the functions and mechanisms of this system in CNS. First, using advanced microscopic imaging techniques and magnetic resonance imaging,^30^ such as two‐photon microscopy, researchers can observe in real time the dynamic processes of astrocytes and the flow patterns of cerebrospinal fluid and interstitial fluid.^20^, ^31^ Second, the role of specific glial cell surface molecules in lymphatic flow is investigated by molecular biology methods such as gene editing and protein labeling.^20^, ^32^ It can also be combined with immunohistochemistry to observe the distribution of cerebrospinal fluid tracers and the expression of specific molecules.^33^ Finally, neurobehavioral experiments were used to assess the effects of abnormal glymphatic system function on animal behavior, such as cognition and emotion regulation.^20^ Almost all of the above methods use medullary tracer techniques, and the combined application of these methods not only enhances our understanding of the structure and function of the glymphatic system but also provides a scientific basis for the development of new strategies for the treatment of related diseases.

### 2.3 Functional features of glymphatic system for the central nervous system

The main function of the glymphatic system is the removal of metabolic wastes from the brain and the transportation of nutrients, and it serves as a bridge between the center and the periphery. The main features are as follows: (1) the direction of fluid transport begins with the entry of cerebrospinal fluid into the periarterial space and then exits along the periventricular space in the “dirty” interstitial fluid; (2) metabolic wastes, Aβ, have been shown to be eliminated through the glymphatic system; (3) fluid transport depends on polarized expression of AQP4 in the vascular terminal peduncle of the astrocytes; and (4) fluid transport and cerebrospinal fluid entry into the neurofibrillary layer are significantly enhanced during sleep, along with an increase in metabolic waste clearance.^31^, ^34^, ^35^, ^36^, ^37^ The glymphatic system also selectively removes selected endogenous solutes such as potassium,^38^ lactate, and pathogenic peptides and proteins (which include Aβ and tau)^31^ and soluble proteins released by damaged cells (neuron‐specific enolase and glial fibrillary acidic protein, among others).^29^

In addition, perivascular exchange of soluble CNS antigens and associated peripheral immune cell interactions contribute to CNS immunosurveillance.^39^, ^40^ It has been shown that the glymphatic system is involved in different processes of neuromodulation such as volume transport, growth factor distribution, and distribution and clearance of inflammatory cells.^41^, ^42^ Decline in glymphatic system function leads to accumulation of large amounts of proteins and blockage of central–peripheral connectivity resulting in imbalance of brain homeostasis leading to aging of the brain and neurodegenerative diseases and cognitive deficits.

The deposition of protein aggregates associated with aging may result from accelerated production or slowed clearance of proteins, or from the introduction of misfolded modular conformations that lead to such aggregation. It was found that clearance of injected Aβ was impaired by up to 40% in aged mice relative to young mice. With decreased CSF‐ISF exchange, vessel wall pulsatility was reduced by 27% in small intracortical arterioles, and perivascular AQP4 polarization was significantly reduced along penetrating arterioles.^37^ Thus, the efficiency of exchange between subarachnoid cerebrospinal fluid and brain parenchyma decreases significantly with age, thereby accelerating brain senescence and aging. It is particularly important to explore the relationship between glymphatic system dysfunction and aging.

## 3 RELATIONSHIP BETWEEN THE GLYMPHATIC SYSTEM AND THE PERIPHERAL IMMUNE SYSTEM

The glymphatic system and the peripheral immune system are structurally and functionally related and interact with each other.^43^ The glymphatic system is the pathway for the flow of cerebrospinal fluid and interstitial fluid supported by astrocytes in the central nervous system and is the main pathway for the removal of metabolic wastes from the brain. The external immune system, on the other hand, is an extensive network of lymph nodes, splenic and mucosal immune systems, and other immune cells that are primarily responsible for immune response, immune surveillance, and fluid homeostasis. They are interconnected and distinct from each other, as shown in Tables 1 and 2.

**TABLE 1:** Correlation of the glymphatic system with the peripheral immune system.

Relevance	Glymphatic system	Peripheral immune system
Immune regulation^44^, ^45^, ^46^, ^47^	The glymphatic system is involved in regulating the central immune response through astrocytes. At the same time, astrocytes can release a large number of cytokines and chemokines, which influence the activity and migration of immune cells.^43^, ^44^	Immune cells in the peripheral immune system, such as T cells and B cells, both communicate with immune cells in the central system through the blood–brain barrier and participate in central immune regulation.^8^, ^48^
Removal of metabolic waste^31^, ^41^	The glymphatic system removes metabolic waste from the brain by exchanging CSF and ISF around cerebral blood vessels through the peduncles of astrocytes.^31^	The external immune system can remove waste and excess fluid from the interstitial fluid of tissues through the flow of lymphatic fluid while participating in the transport of immune cells.
Interactions in pathological states	Dysfunction of the glymphatic system leads to the accumulation of metabolic waste products, which in turn affects abnormal brain function, especially in neurodegenerative diseases.^49^	1. Peripherally released cytokines can cross the blood–brain barrier to cause neurotoxicity and activate microglia and astrocytes.^43^ 2. Peripheral immune cells can participate in the progression of neuroinflammatory and neurodegenerative diseases by infiltrating brain tissue.^43^

**TABLE 2:** Differences between the glymphatic system and the peripheral immune system.

Distinction	Glymphatic system	Peripheral immune system
Anatomical structure	The glymphatic system is a system of cerebrospinal fluid–brain tissue fluid exchange flow mediated by water channel protein 4 on astrocyte end feet.^31^	The peripheral immune system is an extensive network that includes lymph nodes, spleen, and mucosa‐associated lymphoid tissue, among others.
Key features	The glymphatic system is primarily responsible for metabolic waste removal and immune regulation within the brain.^8^, ^30^	The peripheral immune system is primarily responsible for systemic immune response, fluid balance, and pathogen clearance.
Complexity of interactions	The glymphatic system interacts with the peripheral immune system, currently mainly through the blood–brain barrier and migration of immune.^43^	The peripheral immune system involves multiple organs and tissues throughout the body.

Thus, the glymphatic system and the peripheral immune system play an important role in the maintenance of health and disease, and they are interconnected and interact with each other, affecting each other in pathological states. A better understanding of these two systems will provide a new approach to the treatment of immune system dysfunction and neurodegenerative diseases and is also important for the research of aging.

## 4 THE GLYMPHATIC SYSTEM AND THE PHYSIOPATHOLOGIC MANIFESTATIONS OF AGING

### 4.1 Neurodegenerative lesions

**FIGURE 2:**
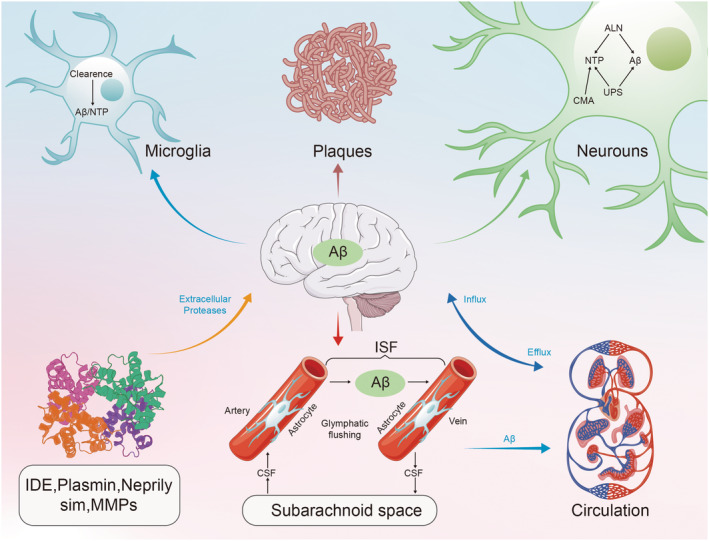
Brain neurotoxicity protein clearance. Neurotoxic proteins (NTPs) are cleared in neurons, glial cells, and vascular endothelial and vascular smooth muscle cells by a series of specific and nonspecific mechanisms. There are three main modes of intracellular clearance of NTPs: (1) the autophagy–lysosomal network (the autophagy–lysosomal network, ALN); (2) chaperone‐mediated autophagy (chaperone‐mediated autophagy, CMA); and (3) the ubiquitin–proteasome system (the ubiquitin–proteasome system, UPS); it is present in neurons, microglia and even other cells. Clearance also occurs in the extracellular space, in the interstitial fluid (ISF) of the brain parenchyma that surrounds the neuron, and in the CSF with which the ISF is exchanged. The extracellular pools of NTP arise from passive diffusion, active release from the terminals, extrusion by cytosolization, and dispersal at cell death. NTP disrupts neuron and synaptic function and is taken up by other neurons and glial cells (“dispersal”). Therapeutically relevant proteases that degrade NTPs include endothelin‐converting enzyme and insulin‐degrading enzyme (IDE) (predominantly cytoplasmic), enkephalinase and matrix metalloproteinases (MMP) (intracellular and extracellular), and fibrinolytic enzymes (mainly extracellular). NPTs that do not enter the neuroglia are driven directly into the peripheral circulation by proteases. The transfer of NTPs to the periphery is regulated by the lymphatic system. Cerebrospinal fluid runs along the periarterial space, crossing perivascular astrocytes carrying water channel protein 4 receptors into the ISF. Arterial pulsation‐driven convection clears NTPs into the cerebrospinal fluid through the neuroglia and perivascular spaces. Glymphatic clearance and cerebrospinal fluid‐driven NTPs enter the peripheral circulation primarily through cervical lymph nodes but also through dural venous sinuses.

As summarized in the literature,^50^, ^51^, ^52^, ^53^, ^54^, ^55^, ^56^ neurodegenerative diseases include AD, PD, frontotemporal dementia (FTD), Huntington's disease (HD), amyotrophic lateral sclerosis (ALS), and tua protein‐related diseases. These protein disorders have complex and unique pathologic and physiologic features, shared by the aggregation of abnormally processed and misfolded proteins.^57^, ^58^ The common feature is the aggregation of abnormally processed and misfolded proteins. The main pathological mechanism is the accumulation of mutated, misfolded, or excessively phosphorylated proteins.^50^ Mutations in Aβ, α‐syn, TARDNA‐binding protein 43 (TDP43), and tau proteins lose their physiological roles and accumulate to create new neurotoxic functions.^57^ These neurotoxic proteins can be cleared from the blood–brain barrier in the glymphatic system in the extracellular fluid, interstitial fluid, and cerebrospinal fluid, on the one hand by enzymatic degradation, and on the other hand by phagocytosis by microglia and astrocytes, which excludes the brain, as in Figure 2.^51^

Age is also a factor that accelerates aging, and the use of MRI to compare changes in the glymphatic system in young, middle‐aged, and elderly people has revealed that the flow of cerebrospinal fluid into the interstitial matrix of the brain decreases markedly with increasing age.^59^ This is consistent with studies in rodents, so is this phenomenon related to the loss of polarization of AQP4 in the end foot of astrocytes. AQP4 deficiency leads to brain aging and produces Aβ aggregation and neurodegeneration. The altered localization of AQP4 may be located upstream of the process of protein mis‐aggregation, suggesting that targeting altered localization of AQP4 may be an effective intervention for Aβ plaques and neurofibrillary tangles, and effective intervention in Aβ plaques and neurofibrillary tangles.^60^ The researchers found that there was a large reduction in AQP4 polarization in certain brain regions in aged mice, such as the striatum and hippocampus,^37^ which further suggests that AQP4 is a key to glial lymphatic system clearance. In the interstitial fluid of the glymphatic system, it was found that α‐syn could flow into the cerebrospinal fluid through glymphatic system clearance, causing impaired glymphatic system function due to reduced AQP4 expression and exacerbating PD‐like lesions in the brains of mice.^61^ Reduced polarization of AQP4 reduces CNS edema.^62^ Impairment of the glymphatic system was found to lead to chronic neurodegeneration and Tua proteopathy in aged mice with IL33 gene deletion.^37^

### 4.2 Immune senescence

Aging of the immune system (called immunosenescence) is also a form of inflammatory aging,^63^ which is a phenomenon in which older individuals express higher levels of inflammatory factors in their cells and tissues, in a state of lower, nonproliferative and chronic pro‐inflammatory response.^64^ Transcriptome sequencing has shown that inflammation‐related genes and signaling pathways are markedly upregulated in aging tissues.^65^, ^66^ At the same time, blood levels of pro‐inflammatory factors such as C‐reaction protein (CRP), interleukin‐6 (IL‐6), and tumor necrosis factor‐α (TNF‐α) are elevated in the elderly.^67^, ^68^ Chronic or excessive release of pro‐inflammatory factors into the CNS results in decreased expression of brain‐derived neurotrophic factor, which is associated with neuroregeneration, glutamatergic activation, oxidative stress, and apoptosis induction.^69^ Studies have shown that T cells undergo changes during immune senescence leading to atherosclerosis, and these cells are thought to be the senescent T cells of SASP.^70^ During the course of chronic heart failure (CHF), increased expression of TLR‐4 by monocytes correlates with increased plasma levels of proinflammatory factors such as IL‐1, IL‐6, and TNF, predicting long‐term productivity in patients with CHF, and similar changes in the TLR system of leukocytes occur with advancing age.^71^ Endocrine disruption may lead to uncontrolled insulin‐like growth factor signaling, aberrant glucocorticoid secretion, excessive androgen production, and insulin resistance, which are involved in the development of metabolic diseases associated with aging.^72^ Immunosenescence is mostly associated with age‐related diseases such as cancer, type II diabetes, cardiovascular disease, neurodegeneration, and geriatric syndromes.^73^, ^74^, ^75^, ^76^, ^77^

The study confirms that enhanced pulsation and compliance of intracerebral arteries in mice reduce neuroinflammation and cerebrovascular dysfunction, leading to improved cognitive performance.^20^ Microglia‐associated inflammatory factors TNF‐α, IL‐1α, and phenyl glycidyl ether (PGE2) activate the glymphatic system to remove metabolic wastes, thereby further regulating extracellular ROS and cytokines.^78^ Recent studies have further emphasized the role of the lymphatic system in the removal of pro‐inflammatory cytokines and chemokines, such as TNF‐α and IL‐1β,^79^ or HIF‐1α in inflammation.^80^

Dysfunction of the glymphatic system leads to elevated levels of inflammation. At the same time, the immune system ages and inflammation levels rise accordingly; low levels of chronic inflammation accumulate to exacerbate the aging process. Both glymphatic system dysfunction and immune senescence lead to elevated levels of the same inflammatory factors such as TNF‐α, IL‐1α, and IL‐6. Therefore, we suggest that glymphatic system dysfunction affects the clearance of metabolic wastes from the brain by the cerebrospinal fluid, which leads to accumulation of metabolic wastes in the brain and disruption of the central immune system accelerating the aging process.

## 5 SLEEP

Sleep is a complex behavioral state and key to maintaining a normal organism.^81^ Recent studies have found that the brain's removal of waste is in a state of high gear during the sleep state.^34^, ^82^ Metabolic waste is removed from brain tissue via ISF and CSF, and sleep plays a key role in the regulation of waste in the brain and the flow of cerebrospinal fluid through the brain.^83^ Compared to awake mice, the glymphatic system cleared up to onefold more during sleep in both natural and anesthetized mice.^58^ Studies have shown that sleep deprivation leads to the accumulation of Tua proteins in the brain.^34^ Neurodegenerative diseases associated with aging have been linked to the accumulation of waste products and proteins in the brain.^84^ These findings demonstrate the importance of sleep and the glymphatic system in aging‐related degenerative pathologies, and that the importance of sleep and the glymphatic system in preventing neurodegenerative diseases of aging may also be the mechanism that leads to the development of these diseases.

A common feature of aging is the progressive loss of circadian behavioral patterns (sleep–wake cycles) and diminished expression of circadian genes. The circadian network regulates various biological processes, and its disruption is not only genetically or environmentally disruptive but also relevant for a variety of diseases associated with aging.^85^ The circadian network regulates a variety of biological processes. The sleep–wake cycle regulates the levels of Aβ‐like proteins accumulated in AD in the ISF and CSF. Further studies found that sleep cycles regulate tau in ISF and that sleep deprivation increases the pathologic spread of tau in ISF and CSF.^83^ Sleep or sleep–wake cycle disorders can lead to dysfunction of the glymphatic system and accelerate the aging process of the organism. The underlying mechanisms behind whether damage to the glymphatic system causes sleep or sleep–wake cycles and thus accelerates the aging of the organism are not clear yet.

## 6 MECHANISMS BY WHICH THE GLYMPHATIC SYSTEM AFFECTS AGING

### 6.1 Chronic inflammation

Chronic inflammation accumulation is an important manifestation of aging, and participating in the aging process is closely associated with the development of aging‐related diseases.^86^ The accumulation of chronic inflammation is an important manifestation of aging. The accumulation of chronic inflammation is facilitated by a number of factors, including ecological dysregulation of the intestinal tract, metabolic abnormalities, changes in hormone levels, accumulation of senescent cells, and aging of the immune system. Senescence‐associated secretory phenotype (SASP) accompanies the increase in senescent cells during the aging process, which further exacerbates the inflammatory response of the body.^87^ In addition, chronic inflammation not only accumulates with senescence but in turn promotes cellular and organismal senescence.^88^, ^89^ High levels of the pro‐inflammatory factors tumor necrosis factor‐α (TNF‐α), interferon‐γ (IFN‐γ), and interleukin‐6 (IL‐6) accelerate the aging process of the organism through autocrine and paracrine pathways.^87^ Inflammatory cytokines such as TNF inflammatory cytokine TNF‐α promote premature aging of rat nucleus pulposus (NP) through PI3K/Akt signaling pathway.^90^

Studying a depressed mouse model of chronic mild unpredictable stress (CUMS) found reduced monoamine neurotransmitter concentrations and upregulation of neuroinflammatory markers in the PFC, reduced intracerebral arterial pulsatility and compliance, and depolarized expression of AQP4 in CUMS mice. The results suggest the glymphatic dysfunction in awake and anesthetized states.^20^ Recently, it was found that CUMS‐induced norepinephrine release reduces AQP4 expression in astrocytes to inhibit glymphatic system function, inducing oxidative stress and inflammation.^91^ The glymphatic system function can influence the changes in inflammatory cells, so whether its alteration has any effect on the changes in signaling pathways related to aging needs to be further investigated.

### 6.2 Mitochondria and oxidative stress

In the 1950s, the doctrine of endogenous free radicals was established, which suggests that free radicals in the body, which are derived from the production of oxygen during basic metabolic processes such as respiration, represent a key driver of aging and aging.^92^ These theories focus on the production of superoxide by mitochondria as a key mediator in the pathophysiology of aging.^64^ High levels of free radicals are generally involved in cellular damage and inflammatory responses, but at low levels, they can also enhance cellular defenses through appropriate stress called “mitochondrial toxic excitotoxicity” (mitohormesis).^93^ Mitochondrial dysfunction in neurons can activate the mitochondrial unfolded protein response (UPR^mt^) in distal tissues, such as the small intestine, suggesting that a number of metabolically regulated circulating factors coexist in multiple tissues.^94^ The mitochondrial unfolded protein response (UPR) dysregulation initiates transcriptional stress in the nucleus, regulating the expression of a range of protein folding, antioxidative stress, and metabolism‐related genes^64^ which leads to organismal senescence.

Oxidative stress is induced by the overproduction of reactive oxygen species (ROS), especially in the brain where mitochondrial activity is most active.^64^ Oxidative stress can be induced by a decrease in the supply of antioxidants and an increase in ROS production in the mitochondria. In studies related to depression, it has been found that dysfunction of the glymphatic system can induce ROS accumulation and pro‐inflammatory signaling, which can damage important macromolecules and induce apoptosis.^95^ Recently, it was found that decreased polarization of astrocyte AQP4 expression inhibits the function of the lymphatic system, thereby inducing oxidative stress and inflammation.^91^ Mitochondrial production of superoxide, which is key to the pathophysiology of aging, also induces ROS and thus oxidative stress, and similarly, impaired function of the glymphatic system induces ROS accumulation. Therefore, we hypothesize that the normal function of the glymphatic system is closely related to it.

### 6.3 Protein homeostasis

Protein homeostasis is an important process that maintains the structure and function of the body's proteins, which is deteriorated with aging^64^ With aging, many proteins become insoluble and accumulate, leading to dysregulation of protein homeostasis and causing processing and folding of neurotoxic peptides in AD, PD, and other proteotoxic diseases.^96^ Decreased function of the glymphatic system also leads to the accumulation of large amounts of proteins and metabolic wastes in the brain, inducing neurodegenerative pathologies and cognitive deficits. Currently, the main pathways that regulate lifespan are related factors that regulate protein stabilization from different levels. For example, insulin signaling can regulate the expression of molecular chaperones and the TOR signaling pathway regulates various forms of autophagy, such as mitochondrial autophagy, a mechanism that removes injured mitochondria from the cell.^64^

In aged mice, the expression of AQP4 was found to be upregulated in tissues surrounding small arteries penetrating the cortex, perivascular AQP4 polarization was absent, and the glymphatic system showed a marked reduction in Aβ clearance.^37^ Comparison of wild‐type mice and mice knocked out of the AQP4 gene revealed that the clearance of Aβ from the brain was reduced by 65% in mice knocked out of the AQP4 gene, suggesting that the majority of soluble protein elimination occurs through the glymphatic system.^31^ Therefore, astrocyte end‐foot AQP4 is the main driver of the glymphatic system, which is extremely important for the clearance of macromolecules and metabolic wastes from the brain.

## 7 SUMMARIZING AND FUTURE PERSPECTIVES

Decreased function of the glymphatic system, which is the main route for removing metabolic wastes from the brain, leads to the deposition of large amounts of toxic metabolic wastes that can damage neurons and glial cells, trigger or exacerbate neuroinflammation, promote oxidative stress, and ultimately lead to neuronal loss and neuronal degeneration, which can induce cognitive dysfunction. Improvement in their function can reverse the course of neurodegenerative diseases and cognitive impairment‐related disorders. Combing through the relevant literature, we believe that the dysfunction of the glymphatic system is the main cause of aging, and its metabolites cannot be excluded from the outside of the brain to be deposited in the brain, which in turn affects the protein homeostasis in the brain, leading to the aging of the organism, and causing neurodegenerative diseases and cognitive disorders. However, the deep‐rooted physiopathological mechanisms of this study are not completely clear, therefore, in the subsequent exploration of the diseases and mechanisms of the glymphatic system and aging, we can pay attention to the metabolic fluctuations triggered by the alteration of neurotransmitters, changes in HPA‐axis hormones, as well as the molecular signaling pathways and neuronal activity in brain regions in the hope that it can provide more ideas for the deep‐rooted physiopathological mechanisms of the glymphatic system and aging and the treatment of the glymphatic system.

In addition, the glymphatic system is closely linked to chronic inflammation, oxidative stress, and sleep–wakefulness. Similarly, immune senescence, chronic inflammation, mitochondrial and oxidative stress, sleep–wakefulness, and protein homeostasis are all important causes of aging. There are fewer studies on the mechanisms related to the glymphatic system and aging, but all of them have something in common. Therefore, we have sorted out and summarized the above mechanisms, and we also hope that the study of the glymphatic system can provide a new therapeutic idea for the treatment of aging and geriatric diseases, and a new direction for the slowing down of aging.

## CONFLICT OF INTEREST STATEMENT

The authors declare that the research was conducted in the absence of any commercial or financial relationships that could be construed as a potential conflict of interest.

## Data Availability

Not applicable.
